# The Application of a Deep Learning Algorithm for the Segmentation of Retinal Nerve Fiber Layer Across Different Optic Neuropathies

**DOI:** 10.1167/tvst.15.4.7

**Published:** 2026-04-10

**Authors:** Roya Arian, Milad Behzadi Far, Reza Sadeghi, Farshad Farnaghi, Mona Safizadeh, Prem S. Subramanian, Neil R. Miller, Yanin Suwan, Pitchapa Kajornrojanaruk, Raheleh Kafieh, Masoud Aghsaei Fard

**Affiliations:** 1 Department of Engineering, Durham University, Durham, UK; 2 Farabi Eye Hospital, Tehran University of Medical Sciences, Tehran, Iran; 3 Departments of Ophthalmology, Neurology, and Neurosurgery, Sue Anschutz-Rodgers University of Colorado Eye Center, Aurora, CO, USA; 4 Neuro-Ophthalmology Division, Wilmer Eye Institute, Johns Hopkins Hospital, Baltimore, MD, USA; 5 Department of Ophthalmology, Ramathibodi Hospital, Mahidol University, Bangkok, Thailand

**Keywords:** retinal nerve fiber, segmentation, optic disc edema, glaucoma

## Abstract

**Purpose:**

To determine the ability of a deep learning (DL) algorithm to segment retinal nerve fiber layer (RNFL) from optical coherence tomography (OCT) scans in glaucomatous optic neuropathies and anterior optic neuropathies with optic disc edema.

**Methods:**

RNFL-Net was developed after preprocessing and automatically removing blood vessels from peripapillary OCT B-scans. It was trained and validated on 1065 RNFL OCT B-scans, and its performance was assessed using 265 test scans. Two different datasets were used for external testing.

**Results:**

The study involved 106 eyes from healthy controls, 118 eyes with optic disc edema, and 60 eyes with glaucoma for training and validation. The segmentation method achieved a Dice coefficient of 0.95 for the validation dataset and 0.92 for test images when compared with manual segmentation. In measuring RNFL thickness in glaucoma-affected eyes, RNFL-Net showed a mean absolute error (MAE) of 6.21 µm and a mean absolute percentage error (MAPE) of 11.24%. The standard OCT device had MAE of 11.05 µm and MAPE of 16.8%. For optic disc edema, the RNFL-Net MAE was 13.04 µm and MAPE 5.71%, whereas the OCT device reported MAE of 22.94 µm and MAPE of 11.2%. For the external validation data, MAE values for glaucoma (*n* = 157) and disc edema (*n* = 32) cases were 7.19 ± 0.14 and 15.41 ± 0.32, respectively.

**Conclusions:**

RNFL-Net can accurately segment RNFL, whereas standard OCT devices produce lower measurements, especially in disc edema.

**Translational Relevance:**

RNFL thickness measurements from RNFL-Net matched the ground truth in glaucoma and optic disc edema cases.

## Introduction

Optical coherence tomography (OCT) measures the thickness of the peripapillary retinal nerve fiber layer (RNFL) using RNFL segmentation—the identification and delineation of the RNFL within images.^1^ This procedure is essential for the measurement of RNFL in various optic neuropathies. In the early stages of specific optic neuropathies, such as non-arteritic anterior ischemic optic neuropathy (NAION), acute anterior optic neuritis (AON), and papilledema, edema and thickening of the RNFL are often observable. In contrast, glaucoma and the chronic stages of NAION, AON, and papilledema are characterized by thinning of the RNFL.^2^^–^^4^

Commercial OCT devices employ specialized algorithms to segment the RNFL by evaluating OCT intensity profiles. Nonetheless, the scans produced by these machines may be influenced by segmentation errors and local defects, such as media opacities, that can result in inaccurate measurements of the RNFL thickness.^5^ For example, in one study, artifacts in RNFL OCT were identified in 58.5% of scans conducted on eyes with glaucoma.^6^ Additionally, posterior RNFL misidentification occurred in 41.7% to 46% of scans analyzed in two other studies.^6^^,^^7^ Of note, these errors in automated segmentation remain relatively stable, and baseline error is highly likely to persist in the same direction and magnitude during subsequent time periods.^8^ In a previous investigation, we found a 64% prevalence of segmentation errors in OCT machines for eyes with previous NAION, resulting in a mean global RNFL thickness that was 6 µm thinner compared with manual segmentation and U-Net algorithm results.^9^^,^^10^

Several studies have investigated segmentation errors in optic neuropathies characterized by RNFL thinning^5^^–^^10^; however, there remains a significant gap in research focused on RNFL segmentation in instances of optic disc edema. Given that inaccuracies in segmentation can influence clinical assessments regarding disease status and progression, accurately segmenting RNFL in various optic neuropathies, whether associated with edema or thinning, presents a considerable challenge.

In light of this, we conducted an analysis of the segmentation errors produced by OCT devices in patients with glaucoma—an optic neuropathy characterized by RNFL thinning—and various optic nerve disorders characterized by RNFL thickening, including acute NAION, acute AON, and papilledema. We hypothesized that employing a deep learning (DL) approach, which provides accurate segmentation of retinal layers, would yield RNFL thickness measurements comparable to those obtained through manual segmentation. Furthermore, we anticipated that this DL method would surpass the performance of current OCT algorithms used for RNFL data segmentation.

## Materials and Methods

### Subjects

This study employed a cross-sectional retrospective comparative design, focusing on eyes affected by moderate to severe glaucoma and eyes exhibiting mild to moderate disc edema attributable to NAION, AON, or papilledema. To design and validate a learning-based supervised system, we collected a dataset from Farabi Eye Hospital, Tehran, Iran, from May 2015 to April 2020. To conduct an external validation of the proposed model, we used two additional datasets derived from distinct populations and ethnic groups. One dataset concerning glaucoma was sourced from Ramathibodi Hospital (Mahidol University, Bangkok, Thailand), whereas the acute NAION data were obtained from the University of Colorado (Boulder, CO). The institutional review board of Tehran University of Medical Sciences and each contributing institution approved the study, and all procedures followed the tenets of the Declaration of Helsinki. Informed consent was obtained from all participants, who then underwent a standard ophthalmic evaluation. We gathered standard ophthalmic evaluation metrics, which included best-corrected visual acuity, demographic information such as age and sex, and spectral-domain OCT (SD-OCT) imaging (SPECTRALIS and HEYEX 6.0; Heidelberg Engineering, Heidelberg, Germany) for the measurement of peripapillary RNFL thickness.

The diagnostic criteria for open-angle glaucoma in the single-center dataset included (1) a glaucomatous optic nerve appearance, characterized by optic disc cupping, neuroretinal rim thinning or notching, and defects in the RNFL as confirmed by glaucoma specialists; (2) an open angle observed during gonioscopy; (3) glaucomatous visual field defects with mean deviation scores less than −6 dB (moderate and severe glaucoma); and (4) thinning of the circumpapillary RNFL on OCT that fell outside the 95% confidence interval of the normal distribution, correlating with the optic disc appearance and visual field defect.^11^ For the external dataset from Thailand, we enrolled cases of mild-to-severe glaucoma. For acute NAION, the criteria included (1) a documented history of sudden, painless vision loss within 1 month prior to enrollment, accompanied by optic disc edema and peripapillary hemorrhage; and (2) visual field defects consistent with an optic neuropathy.^12^ The diagnosis of AON was defined by (1) an episode of painful, subacute vision loss with optic disc edema occurring within 1 month prior to enrollment in individuals 18 to 50 years of age; and (2) evidence of optic nerve enhancement on gadolinium-enhanced magnetic resonance imaging, with or without neuroimaging findings suggestive of multiple sclerosis, neuromyelitis optica spectrum disorder (NMOSD), or myelin oligodendrocyte glycoprotein antibody disease (MOGAD).^9^ Papilledema was defined by bilateral optic disc edema associated with documented elevated intracranial pressure. Regardless of the cause, cases of optic disc edema were excluded if the RNFL thickness by OCT was >350 µm. Patients with other ocular conditions, such as giant cell arteritis, or those with a history of other autoimmune disorders were excluded from all study groups. The control group consisted of individuals with intraocular pressure (IOP) less than 22 mm Hg, no history of elevated IOP, no history of diabetes mellitus, no optic evidence of an optic neuropathy, and no visual field defects by automated perimetry.

### Spectral-Domain Optical Coherence Tomography

For the acquisition of peripapillary RNFL images, the SPECTRALIS SD-OCT was used, capturing two to four images for each study eye. Images with a quality score below 10 were excluded from analysis. The segmentation lines were defined by the conventional OCT software within the SPECTRALIS SD-OCT, marking the anterior and posterior RNFL boundaries in the circular scan, which correspond to the internal limiting membrane and the inner plexiform layer, respectively. The RNFL measurements delineated by the OCT instrument then were recorded. All segmentations produced by the OCT machine were reviewed by an ophthalmologist to identify potential segmentation errors, and manual corrections were applied to the B-scans as necessary. The RNFL thickness data derived from these corrected segmentations were then documented as the “ground-truth” data. In cases where the RNFL could not be identified as a distinct layer, we excluded such images from our analysis, particularly those exhibiting an RNFL thickness greater than 350 µm.

### Peripapillary OCT Data and Annotation

**Figure 1.**
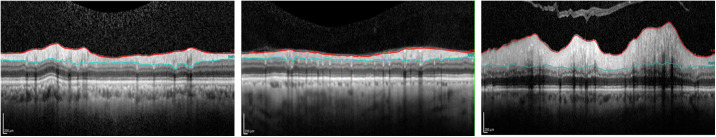
Peripapillary OCT B-scans illustrate a control eye (*left image*), a glaucoma-affected eye (*middle image*), and an eye with optic disc edema (*right image*), each accompanied by their corresponding ground truths for the RNFLs depicted in *cyan* and *red*.

Following the exclusion of 92 scans due to low quality and/or optic disc edema so severe that the RNFL could not be identified as a single layer, the final single center dataset was comprised of peripapillary OCT data from normal control patients (469 B-scans), individuals with optic disc edema (679 B-scans), and glaucoma patients (182 B-scans). Initially, all B-scans were segmented using the standard OCT software in the SPECTRALIS SD-OCT to delineate the upper and lower RNFL boundaries. Subsequently, an expert ophthalmologist (MBF) reviewed all RNFL segmentations generated by the device to identify any inaccuracies. Although some of the device-generated segmentations were accepted as accurate, others contained errors that were corrected manually by the ophthalmologist. For those B-scans with erroneous device segmentations, the annotations made by the ophthalmologist were deemed the definitive ground truth. Figure 1 illustrates a selection of peripapillary OCT B-scans representing each of the three established groups, accompanied by their respective ground truths for the RNFL.

### Train, Validation, and Test Splitting

Initially, 20% of the scans (265 B-scans) with substantial device segmentation errors—defined as gross boundary misplacement or discontinuity of the RNFL borders—were selected from the three groups and designated as the test dataset to evaluate the generalization ability of our models on challenging, previously unseen B-scans. The remaining data were partitioned into training and validation sets using a “subject-wise” method,^13^^–^^15^ ensuring that all B-scans from a single patient were allocated exclusively to either the validation or training dataset to avoid any overestimation of performance. The training and validation dataset was comprised of 1065 RNFL OCT B-scans. Furthermore, fivefold cross-validation was implemented for the three patient groups to facilitate the division of the training and validation sets. In *k*-fold cross-validation, each observation is included in the training set (*k* – 1 times) and the validation set (once), providing a more comprehensive and generalized evaluation compared to random splitting.

### Preprocessing

#### Data Preparation

Because of inconsistency between OCT raw images and RNFL segmented images from the Heidelberg device, RNFL-segmented OCT screenshots were used as the most consistent source. Although these screenshots typically are lower in resolution than direct images, they ensured alignment between the displayed segmentation and the B-scans. This made the process more challenging and necessitated additional preprocessing steps to prepare these screenshots for further analysis.

**Figure 2.**
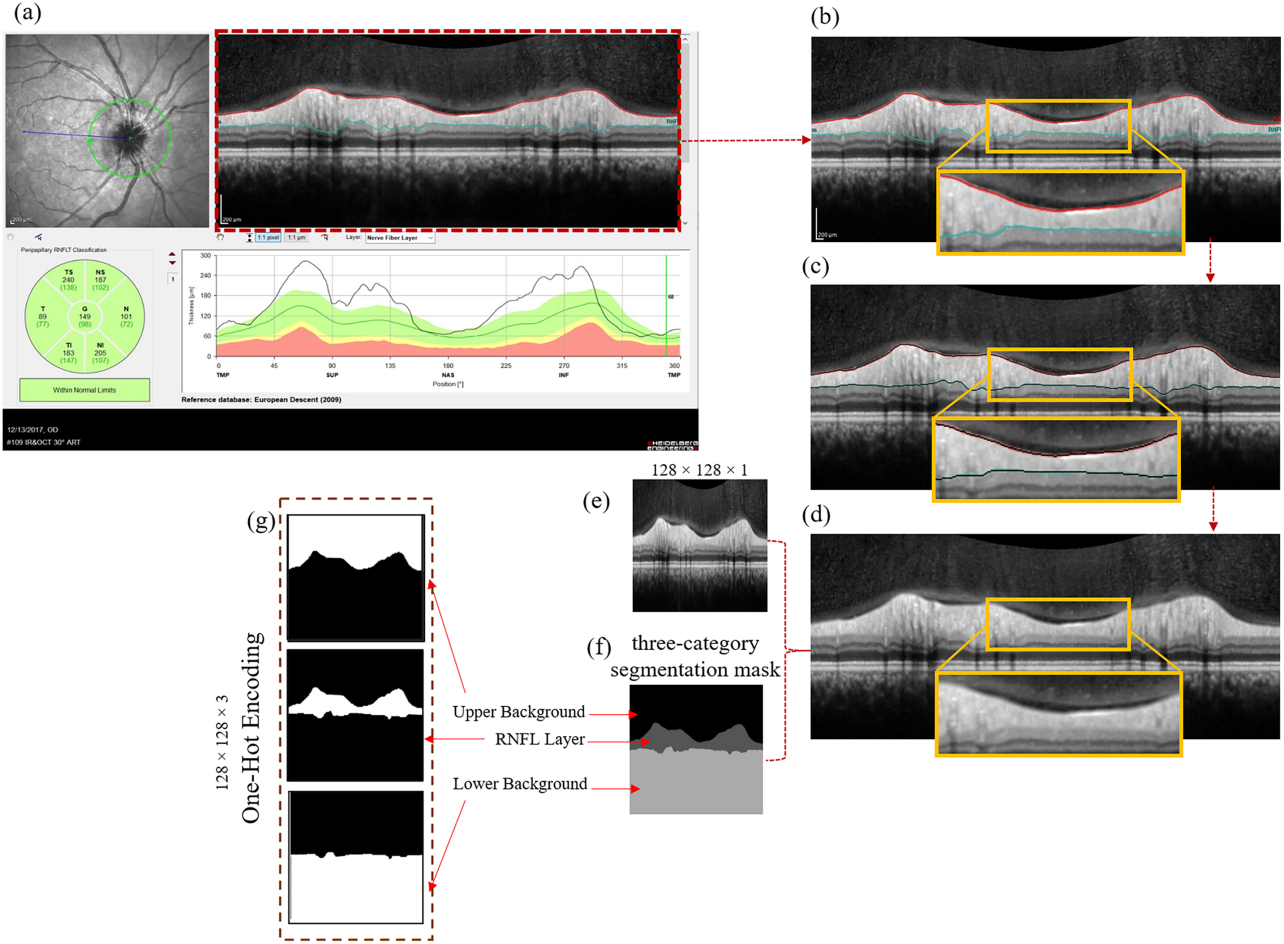
Preprocessing steps of screenshot of B-scans. (**a**) Standard SPECTRALIS RNFL analysis (lower images) and en face (top left) and circle (top right) scans. (**b**–**d**) Delineation of RNFL segmentation with boundary correction and elimination of machine-generated boundary using inpainting. (**e**, **f**) Resized scan analyzed using one-hot coding (**g**) to create the segmentation mask for analysis.

The initial step involved cropping the B-scans from the screenshots. As previously mentioned, the upper and lower boundaries of RNFL were first color-coded on each B-scan by the device software and then reviewed and corrected by specialists if necessary. In this study, these boundaries were identified automatically based on their color characteristics and subsequently removed using an inpainting algorithm. (As the device does not allow export of B-scans without segmentation overlays, inpainting was the only feasible method to obtain images without boundary lines. We acknowledge that this step may introduce potential bias, as U-Net–based models could capture subtle patterns from inpainted regions.) The pixel locations of the detected boundaries were saved and used to generate the ground-truth masks. To reconstruct and fill in missing or damaged areas of an image, one effective method for inpainting involves solving a biharmonic equation, which we employed in this study.^16^ The biharmonic equation was used to reconstruct the masked pixels, matching the analyzed texture and color of the surrounding areas. For this part of the study, we used the detected boundary lines as the mask for inpainting the color-coded boundaries. Next, to create the ground-truth mask for segmenting the RNFL, we designated the area between the ground-truth boundaries as the foreground and the rest of the B-scan as the background. The backgrounds above and below the RNFL exhibit different textures, corresponding to distinct anatomical structures. Consequently, we treated these backgrounds as two separate categories to enhance the ability of the model to differentiate between the various textures, which was particularly challenging when treating both backgrounds as the same. Therefore, we approached our research as a three-category segmentation problem.^17^ Finally, the original B-scans were resized to 128 × 128 × 1 and normalized by dividing each pixel value by 255. Concurrently, the categorical ground-truth masks were resized to 128 × 128 × 3 and one-hot encoded, resulting in binary multichannel matrices where each channel corresponds to a specific category. In each channel, a pixel value of one indicates the presence of the corresponding category, whereas zero signifies its absence. These preprocessing steps are illustrated in Figure 2.

#### Automatic Detection and Elimination of Blood Vessels in Peripapillary OCT B-Scans

**Figure 3.**
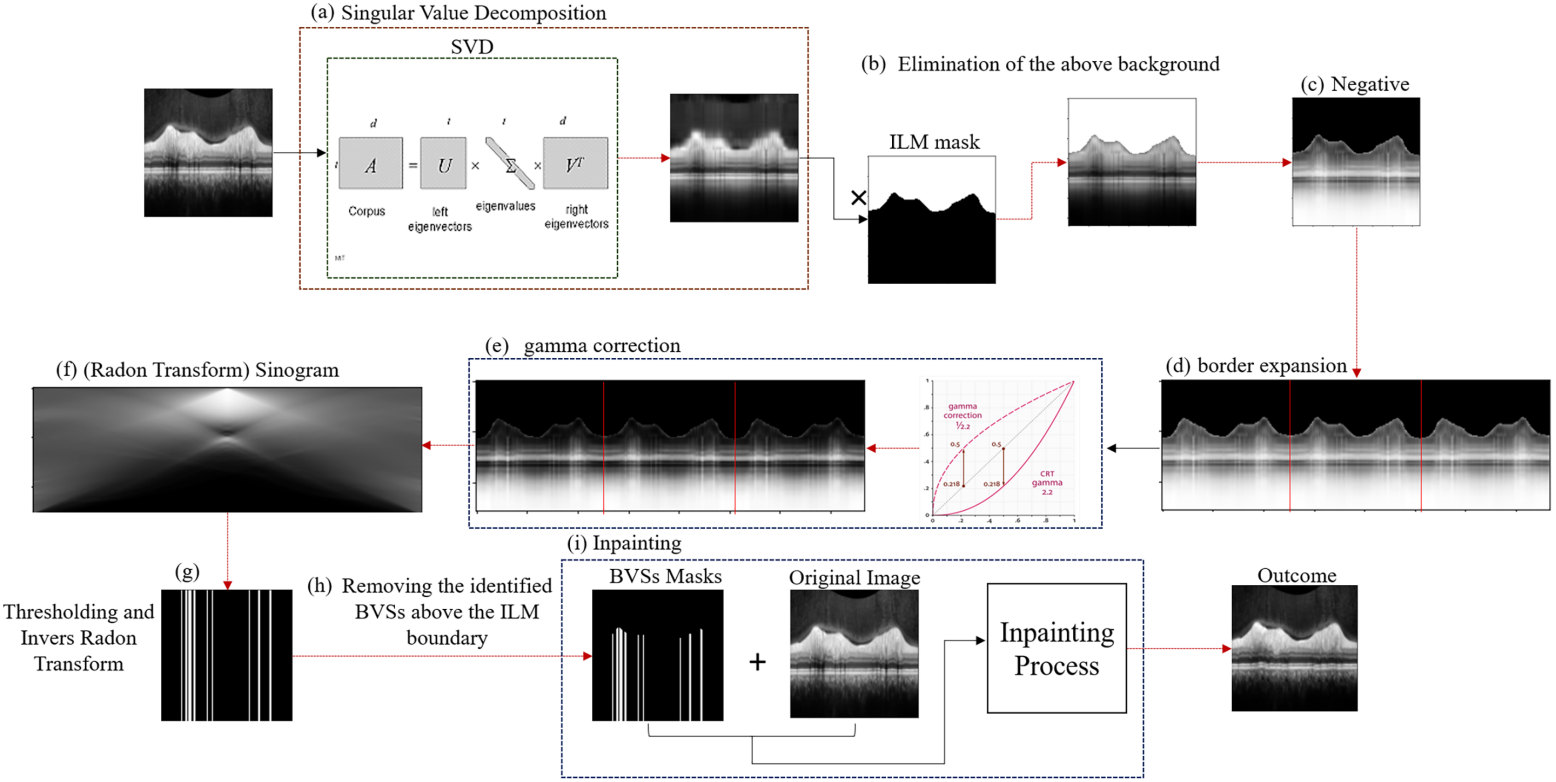
Steps for automatically detecting and eliminating BVSs by integrating SVD.

To remove blood vessel shadows (BVSs),^18^^–^^22^ we used an algorithm and enhanced it by integrating singular value decomposition (SVD) to improve its robustness and reliability.^23^ Moreover, the Radon transform was integrated with SVD.^24^ Figure 3 outlines the steps for automatically detecting and eliminating BVSs. These steps are explained in detail below:

a. In the initial step, SVD is applied to transform each image (B-scan) into a space where vertical lines are accentuated and amplified.
b. To maximize contrast for the BVSs, all pixel values above the inner limiting membrane (ILM) are set to 1.
c. Because BVSs exhibit low gray values that are challenging to enhance, a negative image is created to amplify the visibility of the bright BVSs.
d. The inverse Radon transformation can produce artifacts at the image borders, leading to false BVS detections. This issue arises from the limitations of spatial frequency Fourier transformation, where infinite frequencies are assumed, making accurate reconstruction of border areas in finite images challenging. To mitigate this, the image is expanded by mirroring it on both sides.
e. To enhance image contrast, a gamma correction algorithm with a gamma value of 2.2 is applied, which removes insignificant values that might cause disturbances.^25^
f. The Radon transform then is used to detect vertical lines. Assuming BVSs are the only vertical lines in the OCT B-scans, they appear in the zero-degree column of the Radon space. To facilitate more robust BVSs detection, these lines are reinforced by squaring all values in the first column of the Radon space to enhance contrast, whereas other columns are attenuated using a sloping function. As BVSs might not be perfectly vertical, small angles are less attenuated than higher angles to maintain slightly slanted shadow areas. We found that a sloping exponential function was most suitable for this reinforcement.
g. At this stage, BVSs become identifiable in the trend-regulated gray value summation graph. To detect BVSs and create a BVS mask, a quantile threshold is set at the 85th percentile of the trend data from the graph.
h. Pixels above the ILM layer in the generated BVS mask are set to zero, as these regions are not considered BVSs. BVSs are assumed to originate from the ILM layer and extend downward.
i. Finally, the original B-scan and its corresponding BVS mask are processed using the previously described inpainting algorithm to remove the BVSs.

### RNFL Segmentation

**Figure 4.**
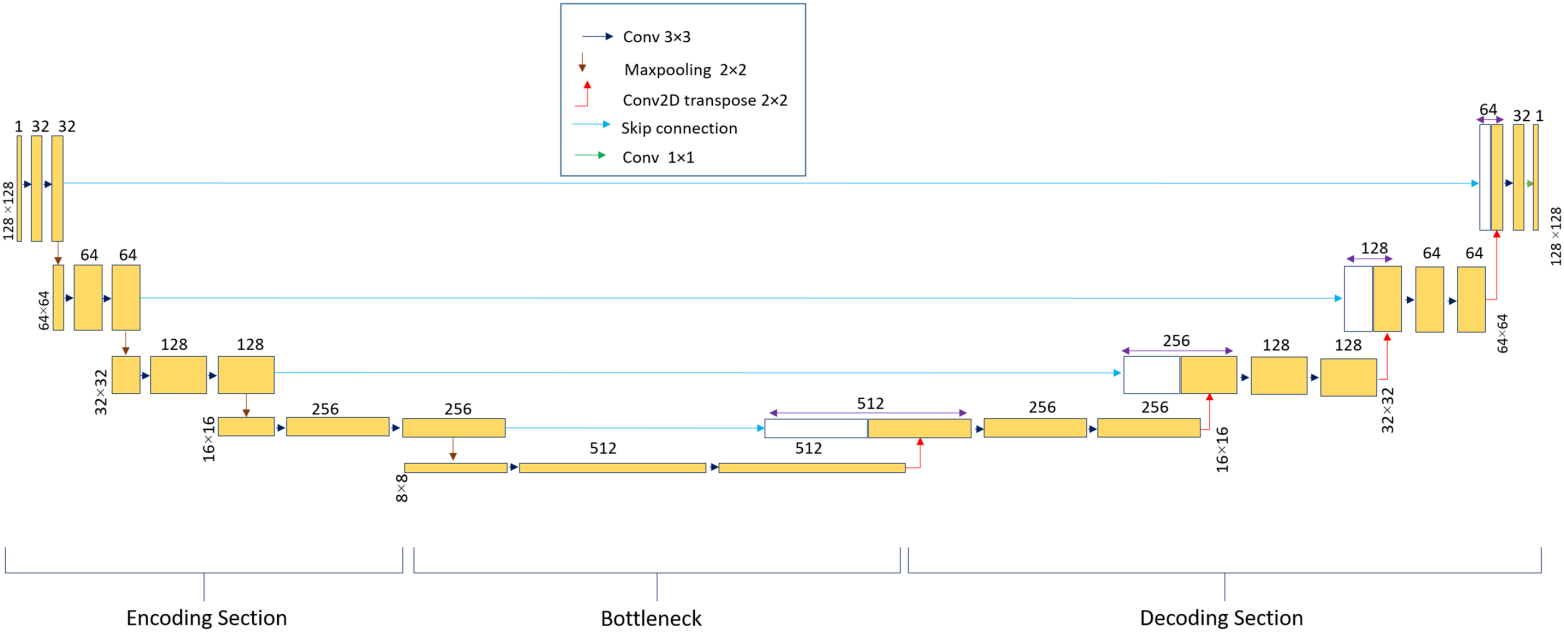
RNFL-Net, a customized U-Net architecture designed for segmenting the RNFL.

In this study, we introduced RNFL-Net, a customized U-Net architecture specifically designed for segmenting the RNFL, as illustrated in Figure 4. This tailored architecture diverges from the original U-Net model by incorporating modifications in the number of blocks, filter sizes, and hyperparameters. The U-Net model, initially proposed by Ronneberger et al.,^26^ features an encoder–decoder framework with skip connections that effectively preserve information during downsampling and upsampling, making it highly suitable for medical imaging segmentation tasks.

The RNFL-Net architecture is delineated as follows: The encoding section comprises four blocks, beginning with an initial filter size of 32, which doubles with each subsequent layer. Each block contains two convolutional layers with a kernel size of 3, batch normalization, and a dropout rate of 0.1. The bottleneck component consists of two convolutional layers with 16 times the initial filter size, totaling 512 filters, a kernel size of 3, and batch normalization. The decoding section also consists of four blocks. Each step in the expansive path involves upsampling the feature map via an upconvolution layer with a stride of 2 and zero padding, which reduces the number of feature channels by half. This upsampled feature map is concatenated with the corresponding cropped feature map from the encoding path. Following concatenation, each block undergoes two convolutional layers with a kernel size of 3 and batch normalization. The final layer utilizes a 1 × 1 convolution to map each 32-component feature vector to the desired number of classes.

Rectified Linear Unit (ReLU) activation functions are employed across all convolutional layers, with the exception of the last layer, which uses a sigmoid activation function. The model is trained with an initial learning rate of 10^−4^, which can decay to 10^−6^ during training, using the Adam optimizer. Additionally, the Dice loss function is utilized as the selected loss metric.^17^ These hyperparameters were determined empirically to optimize loss minimization and achieve low error rates during training.

### Evaluation Metrics

To evaluate the accuracy of the RNFL-Net for segmentation, the predicted RNFL masks are compared with their corresponding ground truths. The performance is quantified using the Dice similarity coefficient (DSC) and the Jaccard coefficient, also known as intersection over union (IoU),^27^ defined as follows: Considering that the RNFL is significantly smaller than the background, DSC and IoU metrics are calculated specifically for the segmented RNFL, rather than for the entire image, to provide a more accurate assessment. The average DSC and IoU values across all B-scans in the test dataset are reported in Table 1.

(1) $$\begin{array}{c} \mathrm{DSC}\left(A,\;B\right)=\frac{2\left(A\cap B\right)}{A+B} \end{array}$$

(2) $$\begin{array}{c} \mathrm{IoU}\left(A,B\right)=\frac{A\cap B}{A\cup B} \end{array}$$

where A represents the predicted masks and B represents the ground-truth masks.

In addition to the DSC and IoU metrics, we also computed the mean unsigned error (MUE) for each boundary by vertically comparing pixel counts between the predicted and ground-truth boundaries. Additionally, we calculated the MAE and MAPE of average RNFL thicknesses across different classes, along with the mean predicted RNFL thickness for various classes and their deviations from true values. These error metrics were evaluated using the test dataset.

All experiments in this study were performed using the Python programming language within a Python 3.7 environment, leveraging the Keras framework. A GeForce GTX 1080Ti GPU (NVIDIA, Santa Clara, CA) was employed for computational tasks. The code and models are accessible at https://github.com/royaarian101/RNFL-segmentation and https://github.com/royaarian101/Automatic-Blood-Vessel-Inpainting.

## Results

### Single-Center Data Collection for Training and Validation

This study included a total of 284 eyes from 117 patients: 106 eyes from 53 normal controls (mean age, 49.5 ± 16.06 years), 118 eyes from 91 patients (mean age, 44.08 ± 14.05 years) who presented with optic disc edema, and 60 eyes from 60 patients (mean age, 53.04 ± 12.01 years) diagnosed with glaucoma. Patients with glaucoma exhibited a significantly higher age compared with those in the disc edema group (*P* = 0.0005, ANOVA). The gender distribution across the three groups revealed a predominance of females in the disc edema group, with the female ratios being 56% in controls, 41% in glaucoma, and 63% in disc edema (*P* = 0.02, χ^2^).The identified causes of optic disc edema included acute NAION in 30 eyes, acute AON in 25 eyes, and papilledema in 63 eyes. In total, 1330 scans were analyzed, consisting of 469 B-scans from control eyes, 679 B-scans from eyes with optic disc edema, and 182 scans from glaucoma-affected eyes. Among these scans, 32.5% exhibited segmentation errors. Specifically, 54 scans from control eyes (11.5%), 258 scans from eyes with disc edema (37.8%), and 121 scans from glaucoma eyes (66.4%) had segmentation errors that subsequently were corrected manually. The training and validation dataset was comprised of 1065 RNFL OCT B-scans, which included 543 scans with optic disc edema, 147 scans with glaucoma, and 375 scans from control eyes. The performance of our algorithm was assessed using 265 B-scans from the test set, which included 136 scans from eyes with disc edema, 35 scans from glaucoma eyes, and 94 scans from normal eyes. Two-center datasets were used for external validation. A glaucoma dataset from Ramathibodi Hospital included 157 mild to severe-glaucoma cases and a second dataset included 32 NAION cases with disc edema from the University of Colorado. Two ophthalmologists from those centers (PK, PS) segmented the OCT images to establish the ground truth.

### Automatic Detection and Elimination of Blood Vessels

**Figure 5.**
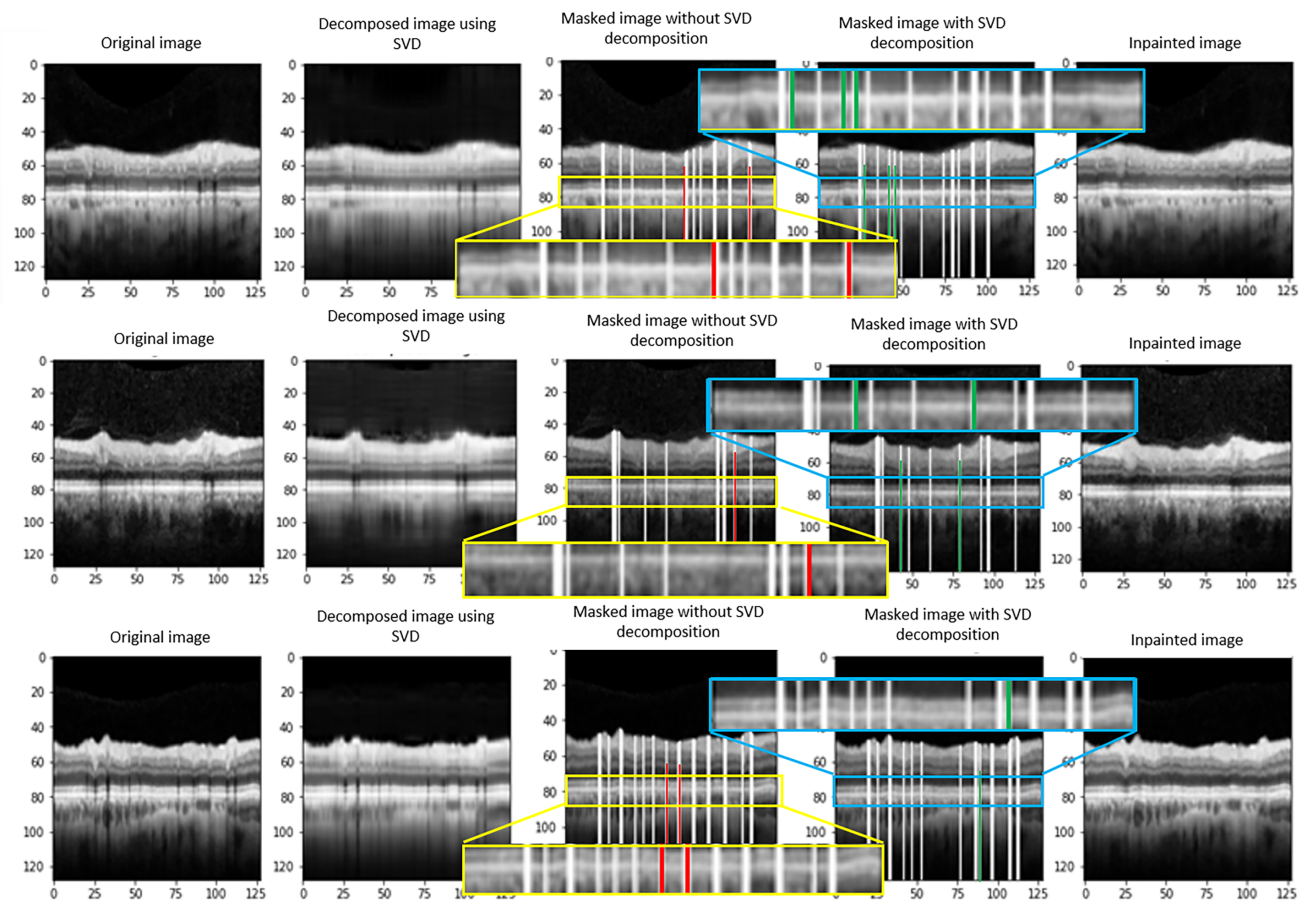
Vessel detection technique: The vessel detection approach that relies exclusively on the Radon transform (*third column*) is compared to our method (*fourth column*), which integrates SVD with the Radon transform. In our combined method, the original B-scans and their corresponding vessel shadow masks are processed using an inpainting algorithm to seamlessly merge the vessels with the surrounding regions (*last column*).

We developed a vessel detection technique that combines SVD with the Radon transform. As illustrated in Figure 5, our method (presented in the fourth column) provides superior vessel detection accuracy compared with the earlier approach (outlined in Reference 24), which relies solely on the Radon transform (shown in the third column). The earlier method sometimes identifies irrelevant vessels or overlooks some existing ones, whereas our technique consistently detects all vessels. Following this, the original B-scans and their associated BVS masks produced by our method undergo processing through an inpainting algorithm to effectively blend the vessels with the surrounding areas (last column).

### RNFL Segmentation and U-Net Performance

**Figure 6.**
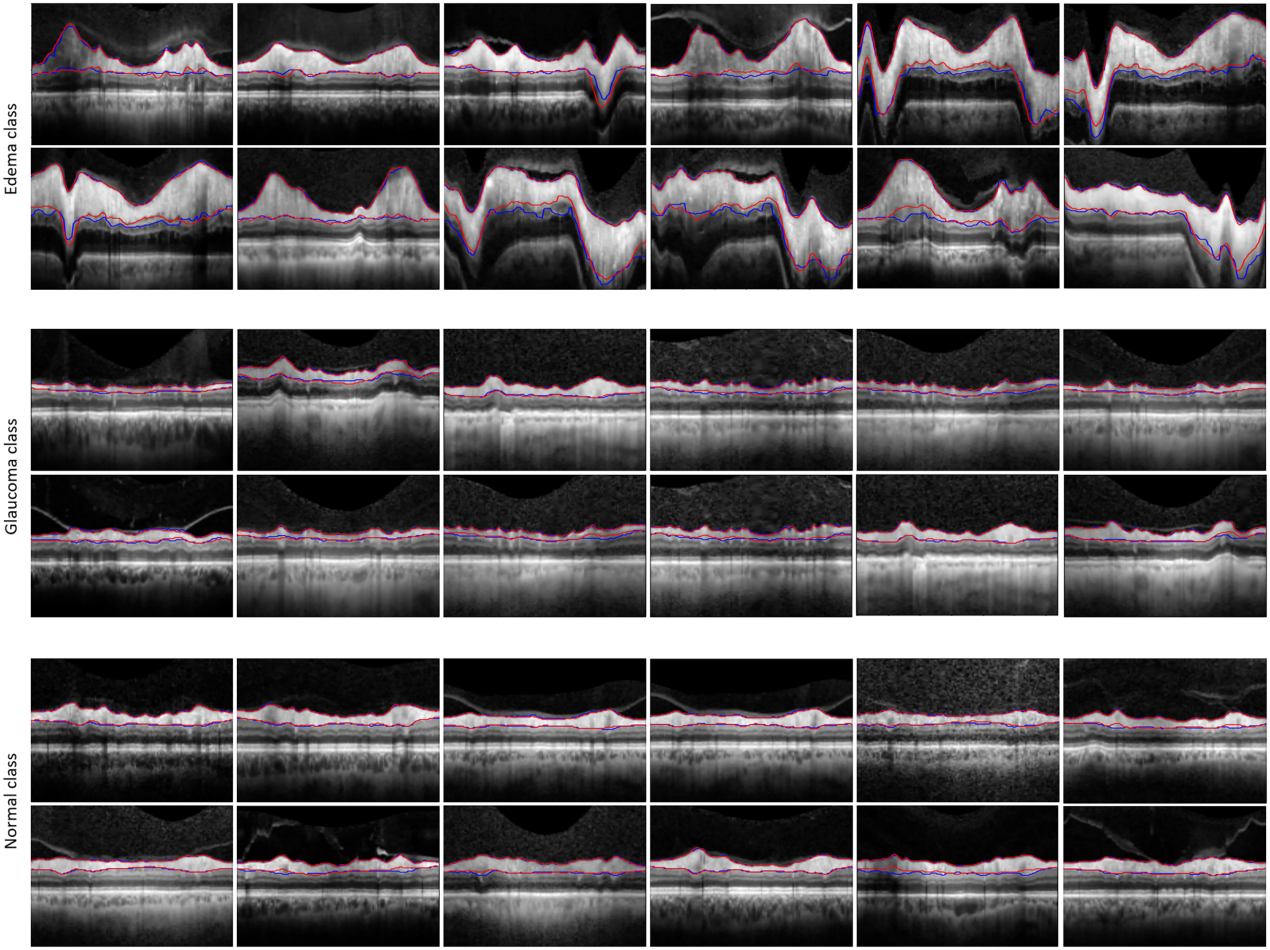
Comparing our network segmentation (indicated by *blue lines*) against the manual ground-truth segmentation (represented by *red lines*) across three distinct classes of optic nerves.

The presence of BVSs in peripapillary OCT B-scans can affect the segmentation accuracy of the RNFL, particularly along the lower boundary. Therefore, RNFL-Net for RNFL segmentation used inpainted B-scans. Figure 6 compares our approach segmentation with manual ground-truth segmentation. Overall, our model yielded high performance in the test and validation images. The sensitivity and specificity of our proposed model on the validation datasets were 0.96 and 0.99, respectively. The same measures on the test sets were 0.93 and 0.99, respectively. The Dice coefficient between our proposed segmentation and manual segmentation by an expert for the validation dataset was 0.95, and for the test images it was 0.92. Table 1 shows sensitivity, specificity, precision, and F1-scores of the RNFL-Net. It is noteworthy that all reported values relate only to the segmented RNFL, not to the entire image.

**Table 1.** Evaluation Metrics of the RNFL-Net Model for the RNFL on Both Validation and Test Datasets

Metric (%)	Validation Dataset (Mean ± SD)	Test Dataset (Mean ± SD)
Dice coefficient	95.92 ± 0.0008	92.87 ± 0.0005
IoU	92.17 ± 0.0015	86.69 ± 0.0009
Sensitivity	96.06 ± 0.0010	93.51 ± 0.0019
Specificity	99.52 ± 0.0001	99.01 ± 0.0002
Precision	95.79 ± 0.0008	92.25 ± 0.0013
F1-score	95.92 ± 0.0008	92.87 ± 0.0005

The reported values pertain specifically to the segmented RNFL rather than to the entire image.

### Average RNFL Thickness in Various Optic Neuropathies

**Figure 7.**
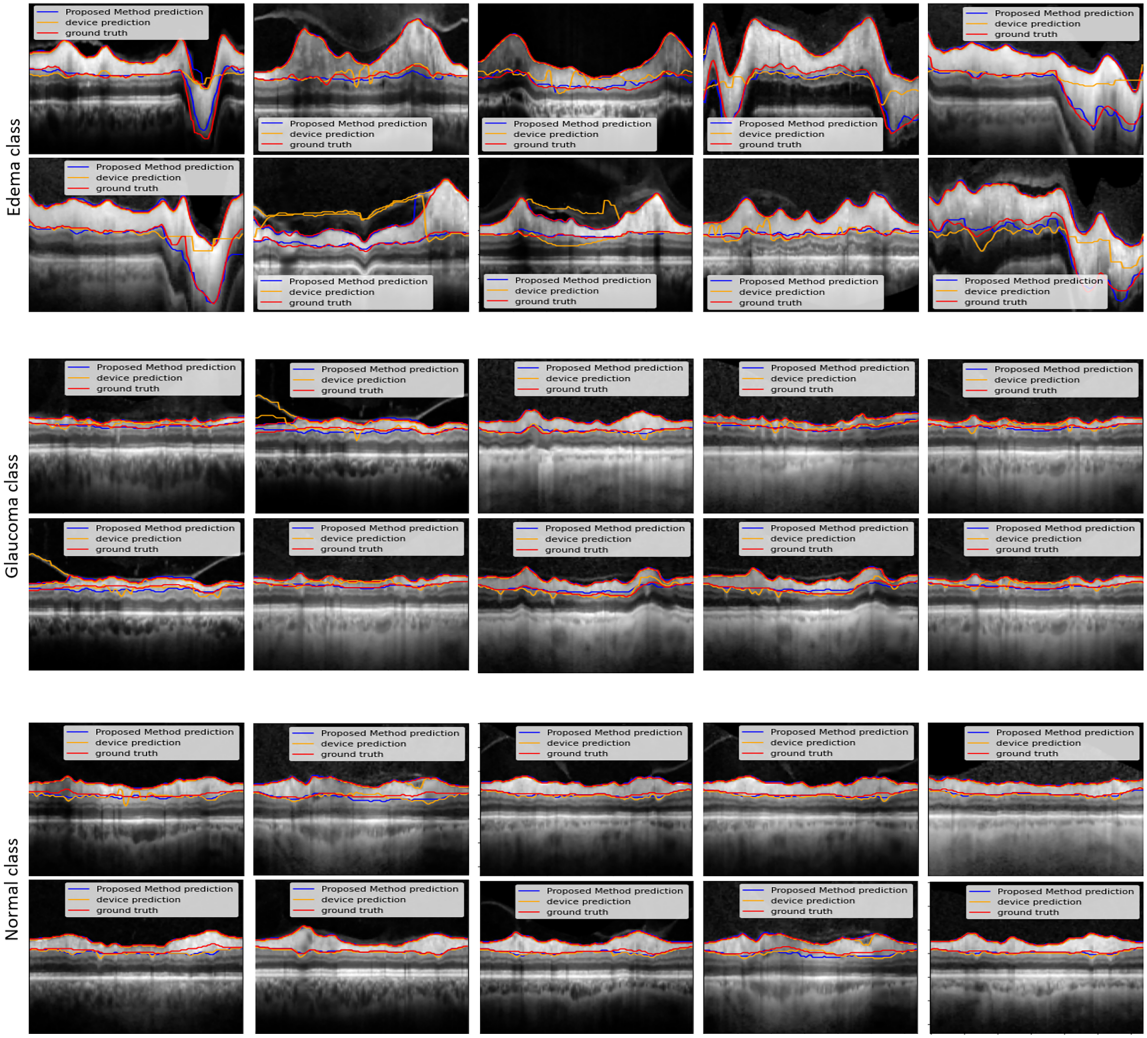
Comparing the performance of the RNFL-Net in segmenting the RNFL with that of the Heidelberg device software and the manual segmented (ground-truth) estimate provided by the ophthalmologist, across normal control eyes, glaucoma eyes, and eyes with disc edema.

Finally, to compare the performance of the RNFL-Net in segmenting the RNFL with that of the Heidelberg device software, we compared the estimate of RNFL thickness measurements by three different methods: our proposed approach, conventional OCT device data, and the manually segmented best estimate determined by the ophthalmologist in the normal control eyes, glaucoma eyes, and disc edema eyes (Table 2; Fig. 7). Overall, the average RNFL thicknesses of normal eyes obtained through our approach and the OCT device were measured at 101.06 ± 0.2 µm and 102.00 µm, respectively. The best estimate from manual segmentation, regarded as the ground truth, was 95.58 µm. In the case of glaucoma-affected eyes, the mean RNFL thickness derived from our algorithm was 67.95 ± 0.50 µm, which closely aligned with the manually segmented ground-truth value of 67.70 µm. In contrast, the OCT device recorded a lower RNFL thickness of 63.69 µm. For eyes with optic disc edema, the mean RNFL thickness was measured at 188.34 µm through manual segmentation, whereas our proposed method produced a value of 188.13 ± 0.81 µm. The OCT device, however, reported an RNFL thickness of 176.9 µm.

**Table 2.** Evaluation Metrics of the RNFL-Net for RNFL Segmentation Using B-scans With Inpainted BVSs as Input

Metric (µm)	Proposed Approach (Mean ± Std)	Device Prediction (Mean)
MUE		
Upper boundary in all classes	**3.99 ± 0.09**	7.98
Lower boundary in all classes	**15.92 ± 0.22**	31.97
Upper boundary in edema class	**5.44 ± 0.10**	11.22
Lower boundary in edema class	**22.18 ± 0.31**	43.35
Upper boundary in glaucoma class	**3.44 ± 0.41**	6.47
Lower boundary in glaucoma class	**11.67 ± 0.56**	17.63
Upper boundary in normal class	**2.09 ± 0.01**	3.94
Lower boundary in normal class	**8.44 ± 0.15**	14.64
MAE thickness		
All classes	**9.74 ± 0.12**	15.06
Edema class	**13.04 ± 0.25**	22.94
Glaucoma class	**6.21 ± 0.13**	11.05
Normal class	6.31 ± 0.13	**4.86**
MAPE thickness		
All classes	**6.73% ± 0.63%**	10.1%
Edema class	**5.71% ± 0.61%**	11.2%
Glaucoma class	**11.24% ± 1.82%**	16.8%
Normal class	6.36% ± 0.23%	**5.5%**
Thickness, all classes		
Predicted	141.20 ± 0.38	135.47
Ground truth	139.32	139.32
Error (difference)	**1.88**	3.85
Edema class thickness		
Predicted	188.13 ± 0.81	176.9
Ground truth	188.34	188.34
Error (difference)	**0.21**	12.34
Glaucoma class thickness		
Predicted	67.95 ± 0.50	63.69
Ground truth	67.70	67.70
Error (difference)	**0.25**	4.01
Normal class thickness		
Predicted	101.06 ± 0.23	102.00
Ground truth	95.58	95.58
Error (difference)	**5.50**	6.42

Better results are highlighted in bold.

To assess accuracy, we calculated the MAE, which represents the average absolute deviation in micrometers, and the MAPE, which expresses these deviations relative to the true thickness. Across all diagnostic groups, RNFL-Net achieved lower MAE and MAPE than the OCT device, reflecting superior absolute and proportional accuracy. Notably, the glaucoma group showed disproportionately higher MAPE despite relatively small MAE values. This discrepancy arises because MAPE is strongly influenced by the magnitude of the reference measurement; in glaucoma, where the RNFL is markedly thinned, even minor absolute deviations yield large percentage errors. Conversely, in disc edema, where the RNFL is substantially thickened, larger absolute deviations contribute to comparatively lower percentage errors. Taken together, MAE and MAPE provide complementary perspectives, as MAE quantifies the magnitude of absolute deviations, and MAPE highlights the proportional challenges of achieving consistent accuracy across conditions with markedly different RNFL thicknesses.

**Figure 8.**
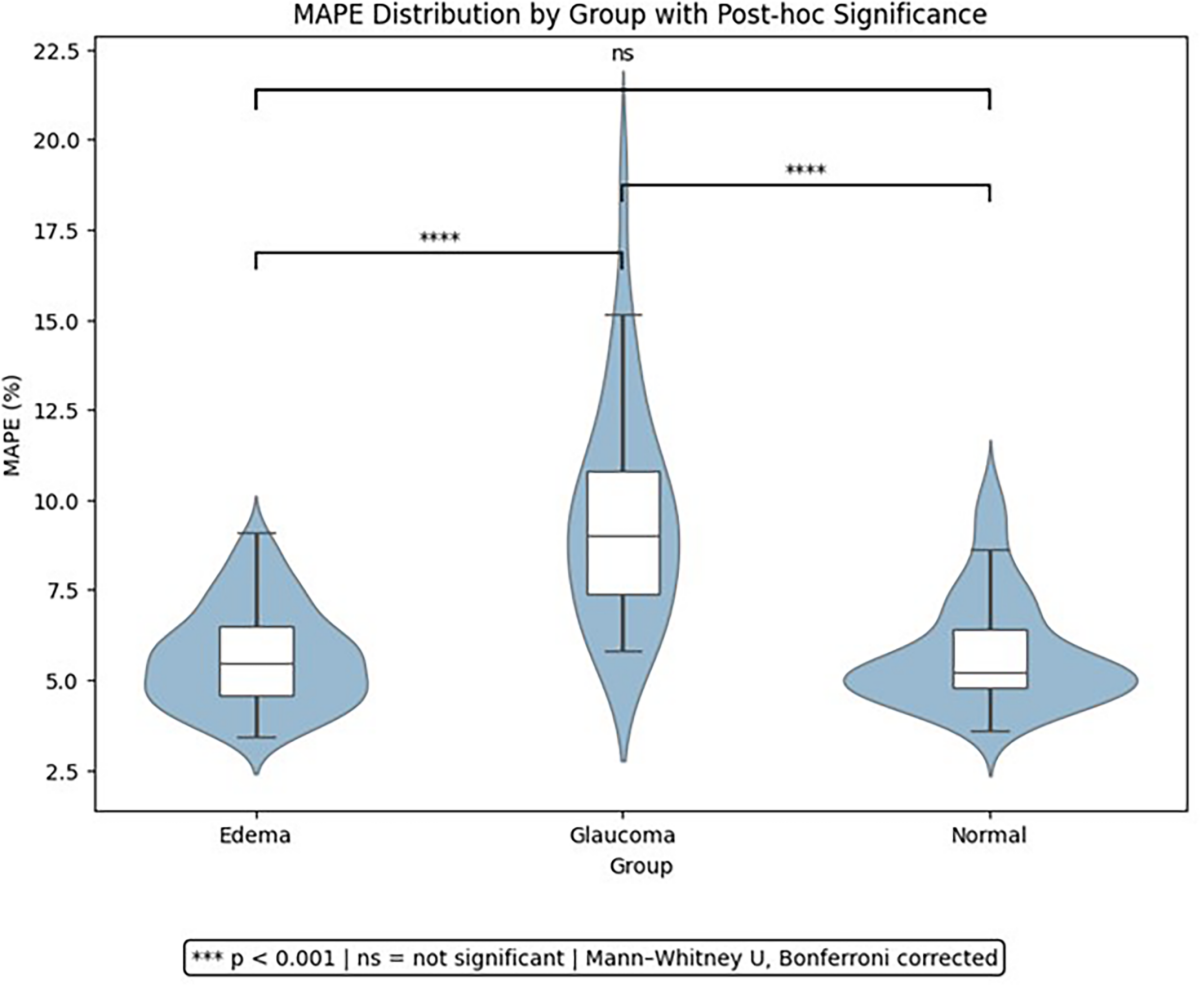
Distribution of MAPE across diagnostic groups (edema, glaucoma, and normal). Violin plots show the full data distribution, with overlaid boxplots indicating medians and interquartile ranges.

To formally compare errors across diagnostic groups, we employed a structured statistical analysis of MAPE (Fig. 8; Table 3). First, we assessed the distributional characteristics of MAPE within each group. Although visual inspection of histograms and *Q*–*Q* plots suggested approximately normal distributions (Fig. 8), Shapiro–Wilk tests revealed significant deviations from normality in all groups (*P* < 0.05), indicating that the assumption of normality was not satisfied (Table 3). Consequently, nonparametric statistical methods were adopted. The Kruskal–Wallis test demonstrated significant overall differences among groups (*H* = 54.3, *P* < 1.6 × 10^–12^). To further localize these effects, we conducted pairwise Mann–Whitney *U* tests with Bonferroni correction. These post hoc analyses revealed that MAPE was significantly higher in the glaucoma group compared with both the edema and normal groups, whereas no significant difference was observed between edema and normal. Collectively, these findings indicate that RNFL-Net maintains proportional accuracy across normal and edematous eyes but exhibits slightly reduced proportional accuracy in glaucomatous eyes, where RNFL thinning amplifies relative error contributions. Although this inflation partially explains the observed differences, it also highlights the inherent difficulty of achieving reliable accuracy in eyes with advanced structural loss characterized by a thin RNFL. Future work should prioritize strategies to improve performance in glaucomatous eyes, such as tailored loss weighting, stratified training protocols, or model adaptations that explicitly account for extreme retinal thinning.

**Table 3.** Statistical Analysis of MAPE for RNFL Segmentation Using B-scans With Inpainted BVSs as Input to RNFL-Net Across Diagnostic Groups

Test	Comparison/Group	Statistic	*P*	Interpretation
Shapiro–Wilk (normality)	All	*W* = 0.845	<0.001	Non-normal
	Edema	*W* = 0.962	0.0008	Non-normal
	Glaucoma	*W* = 0.915	0.0177	Non-normal
	Normal	*W* = 0.873	<0.001	Non-normal
Kruskal–Wallis	All groups	*H* = 54.304	1.6 × 10⁻^12^	Significant
Mann–Whitney *U* (post hoc)	Edema vs. glaucoma	*U* = 367.0	8.8 × 10⁻^13^	Significant
	Edema vs. normal	*U* = 5777.0	0.95	Not significant
	Glaucoma vs. normal	*U* = 2408.0	3.2 × 10⁻^11^	Significant

**Figure 9.**
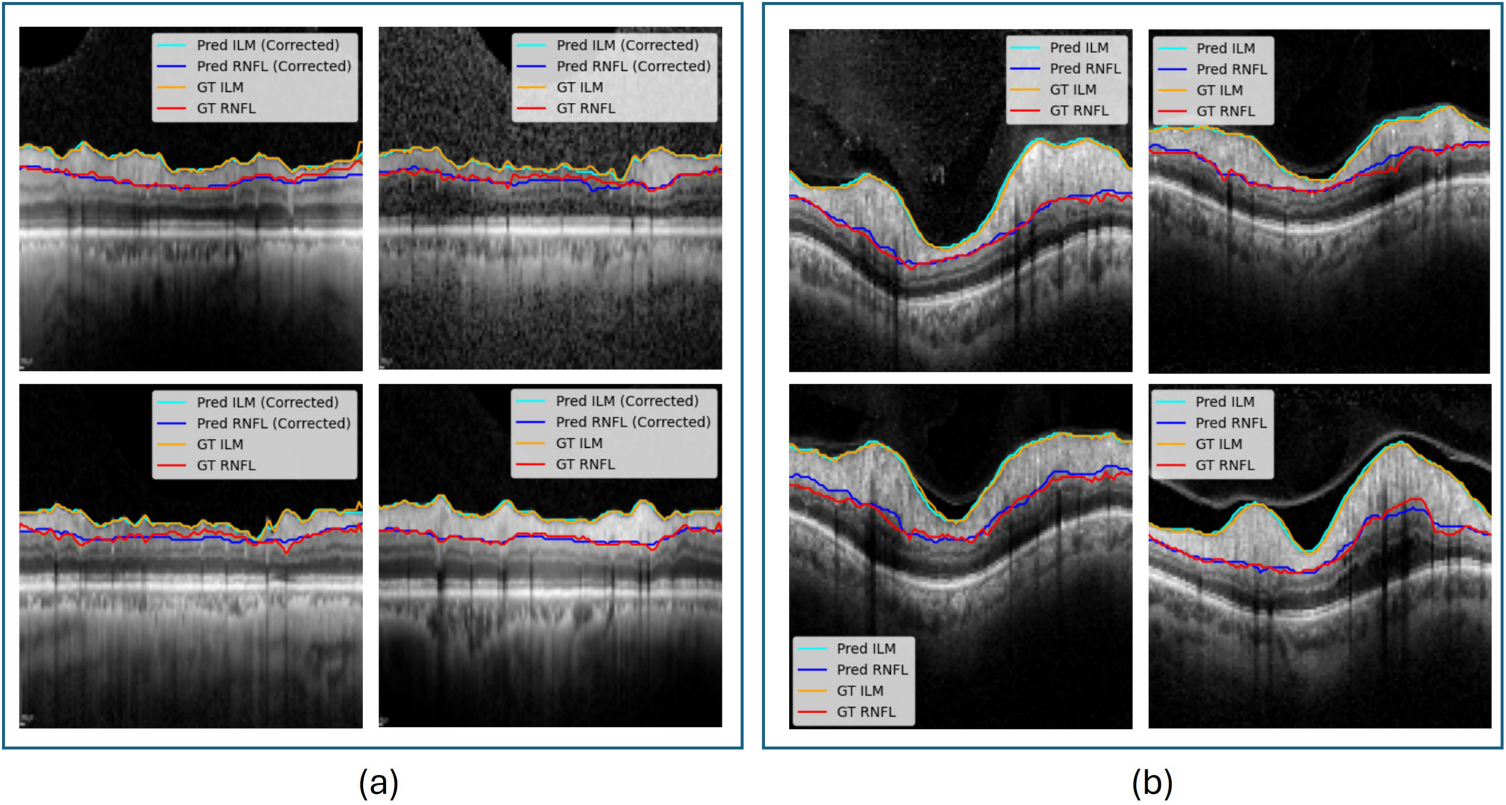
Representative qualitative results from the external test datasets. (**a**) Four sample cases from the Thailand dataset (glaucoma). (**b**) Four sample cases from the U.S. dataset (edema).

To investigate further the generalizability of our model across datasets acquired from different imaging centers and populations with diverse nationalities, we evaluated its performance on two independent external datasets. The first dataset was collected in Thailand and included 157 mild to severe glaucoma cases, whereas the second dataset originated from the United States and consisted of 32 edema cases. These datasets were used exclusively as external test sets, independent from training and validation, to assess the robustness and generalization of our model in unseen clinical scenarios. After integrating the external datasets, two more ophthalmologists from Thailand and the United States helped in segmenting the OCT images to establish the ground truth. The quantitative results of this evaluation are presented in Table 4, and representative qualitative examples are illustrated in Figure 9, demonstrating that the model generalizes effectively across populations and disease phenotypes.

**Table 4.** External Validation Results of the Proposed Model on Two Independent Datasets

Metric	Thailand (Glaucoma, *n* = 157) (Mean ± SD)	United States (Edema, *n* = 32) (Mean ± SD)
Dice coefficient (%)	85.79 ± 0.0019	84.49 ± 0.0008
IoU (%)	75.13 ± 0.0010	73.15 ± 0.0012
Sensitivity (%)	95.79 ± 0.0005	94.66 ± 0.0013
Specificity (%)	99.17 ± 0.0004	98.11 ± 0.0010
Precision (%)	93.82 ± 0.0010	91.83 ± 0.0009
F1-score (%)	94.58 ± 0.0008	93.15 ± 0.0015
Mean MAE (µm)	7.19 ± 0.14	15.41 ± 0.32
MUE of the upper boundary (µm)	6.27 ± 0.10	6.83 ± 0.21
MUE of the lower boundary (µm)	14.52 ± 0.22	24.82 ± 0.40
MAPE (%)	12.73 ± 1.00	6.45 ± 1.12

## Discussion

This study assessed the segmentation and quantification of peripapillary RNFL thickness in OCT images using DL methodologies, particularly the RNFL-Net architecture. The research involved patients with various optic nerve disorders that either thin the RNFL (glaucoma) or thicken it (acute NAION, acute AON, papilledema). Specifically, we performed a comparative analysis between the RNFL thickness estimates obtained through manual segmentation, which served as the reference standard, and those derived from our RNFL-Net algorithm and standard OCT results. We found that our model had excellent sensitivity and specificity rates of 0.93 and 0.99, respectively, on the test datasets. Additionally, the RNFL thickness measurements obtained from the RNFL-Net segmentation were consistent with the manually derived values (considered the ground truth) in both glaucoma and optic disc edema cases. In contrast, the data from conventional OCT devices were lower in these two groups and exhibited significant segmentation inaccuracies. However, in normal controls, where segmentation errors were minimal, the values from our approach aligned closely with those from the OCT.

Errors in the segmentation of RNFL and the evaluation of its thickness previously have been noted in a range of optic neuropathies, including glaucoma and post-acute NAION, with prevalence rates ranging from approximately 46% to 62%.^7^^,^^10^ In this study, we found that 32.5% of the scans exhibited segmentation errors, with a notable 37.8% error rate in eyes with optic disc edema and a 66.4% error rate in glaucoma-affected eyes. Furthermore, our findings revealed that device-automated segmentation yielded a thinner average global RNFL thickness in glaucoma cases, specifically 4 µm less, than that obtained through manual refinement. Similarly, research by Mansberger et al.^5^ indicated that data from automated OCT machines resulted in an RNFL thickness measurement that was 1.6 µm thinner than the reference measurements. More importantly, our study found that OCT machine assessment of RNFL thickness in instances of optic disc edema was 11.4 µm less than the manual ground-truth measurement. Therefore, the manual refinement of RNFL images in cases of disc edema is more critical than in glaucoma cases, as the degree of underestimation is considerably greater.

Various methodologies can be employed for RNFL segmentation,^28^^–^^30^ including (1) image processing techniques, such as thresholding, which uses intensity values to differentiate the RNFL from adjacent layers; (2) machine learning approaches, including supervised learning with algorithms such as support vector machines (SVMs), random forests, and neural networks, which are trained on labeled datasets to classify pixels as RNFL or non-RNFL; (3) OCT-specific techniques that use specialized algorithms to segment the RNFL by examining OCT intensity profiles; and (4) DL techniques, where convolutional neural networks^31^ serve as powerful tools for semantic segmentation, capable of learning complex features from data to accurately segment the RNFL. The U-Net architecture is particularly effective for RNFL segmentation due to its encoder–decoder structure. Several investigations have examined the application of DL algorithms to address the complexities of OCT layer segmentations. For example, Devalla et al.^32^^,^^33^ created a DL algorithm specifically for optic nerve head scans that exhibited notable accuracy when juxtaposed with manual segmentation techniques. This research group also presented a 3D segmentation framework (ONH-Net) that can be effortlessly adapted to various OCT devices for both healthy and glaucoma-affected eyes. Although their research did not assess RNFL thickness, they successfully automated the segmentation of OCT volumes from a novel device without the necessity of retraining ONH-Net using manual segmentations from that particular device. Similarly, Yow et al.^34^ used U-Net for RNFL segmentation and quantification, employing cross-sectional OCT scan images to generate a binarized circumpapillary RNFL mask. Regarding the clinical applications of DL, Jammal et al.^35^ developed an algorithm capable of identifying RNFL segmentation errors in both glaucoma and normal eyes. Their study included a test sample consisting of scans with at least one RNFL segmentation error, as determined by a human grader, and the algorithm was trained to predict the likelihood of segmentation errors in the test dataset. With a probability threshold of 0.5, the DL algorithm achieved a sensitivity of 95.0%, accurately identifying 1172 of 1234 scans that contained segmentation errors. In a different study,^36^ the same research group predicted RNFL thickness from raw, unsegmented scans using DL methodologies. They found a strong correlation between segmentation-free DL RNFL predictions and conventional OCT RNFL thickness measurements in the images.

In light of the above reports, as well as our own findings, it is clear that quantification and segmentation of the RNFL is essential not only for eyes with glaucoma but also for eyes with other optic neuropathies. In this study, we performed RNFL thickness measurements through segmentation across a spectrum of optic neuropathies, encompassing both RNFL thinning and thickening, which distinguishes our research from previous studies. In this research, we implemented a series of DL techniques for segmentation of the RNFL. During the data preparation phase, we employed several innovative methods. Initially, we used an inpainting algorithm to eliminate the color characteristics associated with the boundaries of the RNFL. This step is crucial as it allows the model to concentrate on the genuine features of the RNFL, thereby ensuring precise segmentation without the interference of color-coded boundaries added by specialists. In addition, inpainting serves to reconstruct and fill in areas of an image that are either missing or damaged, leveraging information from adjacent, intact regions. A particularly effective inpainting method involves solving a biharmonic equation,^16^ which guarantees that the filled areas are smooth and seamlessly integrated with the surrounding image regions. The process commences with the creation of a mask to identify the damaged or absent areas. Subsequently, the non-masked regions are analyzed to capture their texture, color, and structure. Following this preprocessing, we introduced a novel technique for the automatic detection and removal of blood vessels in peripapillary OCT B-scans. In these B-scans, blood vessels manifest as vertical dark shadows, which can compromise segmentation accuracy, especially in identifying the lower boundary.^18^^–^^22^ To address this challenge, we used an automatic blood vessel detection method based on SVD.^23^ We further refined this algorithm by incorporating SVD to enhance its robustness and reliability.^23^ When applied to images, SVD aids in isolating significant features, such as vertical lines, by concentrating on the principal components derived from the decomposition process. In this study, SVD successfully highlighted vertical lines, likely representing blood vessel shadows, while simultaneously smoothing and blurring areas of lesser significance. Additionally, the Radon transform^24^ functioned on line integrals, which effectively improved the identification and enhancement of vertical vessel shadows, and this method operates independently, eliminating the need for additional models, training, or supplementary data. Ultimately, this study found that RNFL-Net, a tailored U-Net architecture specifically created for the segmentation of the RNFL, proved superior to the other methods that we assessed.

Our study had several limitations. First, our dataset was smaller than those typically used, such as those used in pure glaucoma research, which is not surprising given the relative infrequency of optic nerve disorders associated with disc swelling compared with glaucoma. Nevertheless, it is possible that a larger database with more optic nerve disorders characterized by optic disc edema might have given different results. Second, we did not have proper alignment between the exported raw B-scans and the corresponding device-generated segmented images, which made direct use of raw data unreliable. To address this, we used RNFL-segmented OCT screenshots as the most consistent source, and applied inpainting to remove the segmentation overlays. This necessary step may have introduced bias, as U-Net–based models can capture subtle patterns from the inpainted regions. In future work, access to properly aligned raw B-scans without overlays will be essential to eliminate the need for inpainting and reduce the risk of such bias. Additionally, our U-Net model was specifically trained using images from the SPECTRALIS OCT machine, potentially limiting our ability to apply our algorithm to scans obtained from other OCT devices. Although we mitigated this by validating the model on two independent external datasets from Thailand and the United States, future work should expand to multicenter, multiple-device training cohorts and substantially larger datasets to further enhance robustness and generalizability. Finally, our supervised DL algorithm was designed to segment the RNFL in cases of mild to moderate disc edema, and it is essential that improved imaging techniques and algorithms are developed in the future for severe disc edema cases.

Through the application of RNFL-Net, we segmented the RNFL in a range of optic neuropathies, including edematous discs and optic disc atrophy. The RNFL thickness measurements derived from the RNFL-Net segmentation demonstrated a consistency with the ground truth in cases of glaucoma and optic disc edema, whereas standard OCT devices reported lower measurements in these two categories and displayed considerable segmentation inaccuracies. It is evident that RNFL-Net surpasses the Heidelberg device software in segmenting the RNFL, particularly in detecting the lower boundary, resulting in a MAE of predicted thickness across all classes.
